# A Letter from Paris

**Published:** 1875-07-01

**Authors:** C. C. Matteson

**Affiliations:** Paris


### A LETTER FROM PARIS.

EDITORS MEDICAL EXAMINER: The Parisians are very fond of journals. Those secular are in myriads, while those devoted to medical subjects may be counted by dozens. One can have a fresh medical journal every day, Sundays included, and some days many appear together. There seems to be no established rule for time of publishing. Certain journals make their appearance every three months, as the *Revue des Sciences Medicales;* others come every two months, as the *Archives de Physiologie*, while there are innumerable monthlies and several semi-monthlies, as for instance, the *Journal de Therapeutique*. Then there is an "every ten days" paper named the *Journal des Connaissances Medicales Pratiques et de Pharmacologie*, and we find eight or ten weeklies, among which the *Gazette Hebdomadaire de Medicine et de Chirurgie* occupies a prominent position. Shortening the dates we next arrive at the *France Medicale*, which is original in appearing twice a week. Finally we reach the *Gazette des Hopitaux* and the *Union Medicale*, which are published three times a week. My "finally" was perhaps a little too positive, for there is a journal, the *Bulletin de la Medicine et de la Pharmacie Militaires*, that is announced "to appear at irregular dates, whenever important matter necessitates its publication." As far as quantity goes there is nothing to complain of, but, unfortunately, one can see very plainly that a consolidation of many of the now separate publications would be a great boon to the profession. The difficulty seems to be that each man of any prominence must have a journal of his own which he can control and which shall be devoted to his own ideas. This way of working of course makes the number of journals great, but each one, more or less dependent upon the writings of its owner, is obliged to be filled up with copied articles. But let us look over some of these journals. Here, for example, is the *Archives de Tocologie*, including the diseases of women and children, which is edited by M. Depaul, Clinical Professor at the Paris school. The reputation and vast experience of the

Professor insure a certain number of valuable and instructive articles, but one hardly cares to subscribe for a journal largely dependent on the writings of one person. On the same subject there is another publication, also monthly, the *Annales de Gynæcologie*, which is edited by Messrs. Pajot, Courty, and Gallard. The objection of personality does not apply so strongly to this journal, although one naturally regrets that it lacks the support of the clinical professor. To keep well posted one is thus necessitated to subscribe for both journals. Another branch is well represented by the *Bulletin General de Therapeutique* of Professors Behier, Bouchardat and Dolbeau, and by the *Journal de Therapeutique* of M. Gubler. Each of these journals is as a rule well supplied with original and instructive articles, and the latter, though only in its second year, is justly popular. M. Gubler is regarded as possessing a thorough knowledge of the state of therapeutics at the present day, and his articles and lectures on this subject are certainly without superior in France. However, the necessity for his enlarging the number of journals is not apparent, and the objection of personality still holds good. To get his articles one must make room on his already crowded table for another journal. Viewing the mass of publications, the wish is only too natural that the good might be condensed into half a dozen, or indeed a dozen, journals, as in this way might be saved a vast amount of paper, time, and patience, to say nothing of the smaller item of money. But to continue, students are rather partial to the *Progres Medical*, a weekly that gives considerable space to information on changes in the hospital services, and such other news as students are interested in. Mentioning this journal reminds me of a great difficulty that a stranger always meets with in visiting the Paris hospitals. The buildings are so extensive, and the wards so numerous, that it is almost impossible to find any particular service without a guide. There is no bulletin board to aid one, and many times the visit is made and the morning wasted without one finding the object of his search. The *Progres Medical* has had the enterprise to publish, at the opening of the winter session, what is called a "student's number," in which the location of each hospital is printed, with the names of the attending physicians and surgeons, the names of each of their wards, and also, as far as possible, the days and hours of lectures. With this in hand, one can make a hospital visit with some confidence. By procuring a copy of this number, at the office of the journal (6 Rue des Ecoles), the medical visitor will save much time, as I can guarantee from sad experience. Most every one reads the *Gazette des Hopitaux* or the *Union Medicale*, the two tri-weekly journals. The former has under its proper title the words, "La Lancette Francaise," but it is rather an insignificant affair when compared with its London rival (sic). However, this journal has a very firm footing, and occupies a prominent position. As its name shows, the articles that it contains are on cases and lectures of the hospitals, and, though not too prompt as to time, one can get a very fair idea of what is going on in the public service of Paris by reading this paper. The *Gazette des Hopitaux*

is reliable, and all its editorials are conservative, as representing, so to speak, French medical opinion authoritatively. Of course, with so many medical publications, there is a great demand for material to print. One subject is common property—that is, the reports of the various scientific societies. Nearly every number of each journal gives a goodly space to these reports, and the editorial page is generally devoted to consideration of discussions in the Academy. The journals that appear at longer intervals as a rule have one or more original communications, but under this head are found quantities of articles by the internes of the hospitals. The weeklies and tri-weeklies are sometimes hard pressed for material, at least apparently, and their pages are filled with essays that would be interesting to the general public. This is not so bad a way after all, for one finds in his medical journal interesting reading on history, travels and events of the day. The *Union Medicale* has a "Feuilleton" that nearly always contains some amusing and well-written article, though the choice of subjects is very varied. One day we find an account of transactions in the French school in 1600 or 1700; another day an essay on women-doctors, while in the number I have before me there is a criticism on the paintings in the exhibition that is now open (Salon of 1875). The writer keeps in mind that his article will be read by medical men, and therefore treats his subjects from a medical stand-point. He criticizes the portraits of physicians, of which there are many in the exhibition. Nor is the writer mild in some of his criticisms. Mentioning the portrait of a certain French doctor, he writes: "It is signed by an American name, which I prefer not to mention so that I may be more at ease in saying how horrible I find this portrait." Then he discusses the particular defects that he finds in this subject. However, he does not limit his severity to the work of the American, but writes of one work of art as follows: "Another painting, numbered 337, and marked 'Venus,' is interesting to pathologists. This large woman, red and naked, but whose nakedness does not seem to embarrass her, is troubled with several diseases, the nature of which it is possible to determine approximatively. The swelling that deforms the right thigh, and seems to place the buttock as high as the loin, the turning inward of the lower limb of the same side, and especially of the foot, indicate perfectly that there is a coxo-femoral luxation of the ilio-ischiatic variety. The left leg, on the contrary, is arranged in such a way that the inner side shows to the front while the thigh of the same side rests in the normal position. Was there a luxation of the knee of this side which has left after it this singular change in the respective position of the vertical axes of the two segments of the lower limb? These lesions are doubtless explained by the lymphatic character of this unhappy Venus." In this style he reviews the works of art in the exhibition, and his criticisms are generally very just, as any one would admit after viewing the paintings.

The general appearance of the medical journals of this city is by no means handsome, and everything seems arranged with a prominent idea for economy. Yet the subscription price is not, as a rule, low, and one has to pay from fifteen to thirty francs (three to six dollars, gold) a year for each journal, which is proportionally much more than we pay in the United States, where the expenses are so much greater. French journalism, whether medical or secular, is "sui generis," and is not imitated by other nations—shall I add, fortunately?

When Professor Pajot launched his thunderbolt against the use of anæsthetics in natural labor, it seemed as though the quietus had been given to discussions on this subject among the physicians of France, but now there appears in the *Annales de Gynæcologie* for May an article by Dr. Chouppe on the advantages of using the hydrate of chloral in labor. The writer starts off with an half-apology for writing on this subject at all after the essays of M. Campbell and Prof. Pajot (particularly the latter's), and states that he does not intend to discuss anæsthetics in general, but merely desires to show the most recent experiences with the chloral under the circumstances mentioned. He gives to M. Ore and to Prof. Vulpian the credit for demonstrating the anæsthetic power of chloral, although he admits that England, "the country of obstetrical anæsthesia," was the first place where chloral was given during labor. An examination of the dates that he records—1870, the year when Dr. Lambert first *published* his results in Edinburgh; 1872, the year of the first *trial* in France, and 1873, 1874, the years of Messrs. Ore and Vulpian's experiments—such an examination shows that France has as little to claim for this procedure as for other anæsthetical advancements. Dr. Chouppe gives at length the history of twelve cases, five of which appear for the first time. Following these histories is a table of thirty-seven cases collected from different sources. Among other data this table records the condition of the os uteri when the chloral was given, the amount of the drug that was exhibited, and the results of its use. In twenty-nine cases the anæsthesia was complete after the use of from two to eight grammes of chloral. Three cases are recorded as showing no signs of anæsthesia after doses, in the respective patients, of six decigrammes, four grammes, and eight grammes. "Prolonged anæsthesia" was produced in two patients after the use of twelve and sixteen grammes, while the other cases are recorded as having shown a momentary or partial anæsthesia. To prove that chloral does not interfere with uterine contractions, Dr. Chouppe gives the details of one experiment on a bitch. The abdomen having been laid open, so that the uterus could be easily examined, the contractions of the latter were tested, and then two grammes of chloral were injected into the intestine. Contractions of the uterus remained as before, and the same was the case after a second injection of two grammes, although the animal was completely insensible.

In a long resume of the question, the writer announces that chloral gives relief from pain to the woman in labor without interfering with uterine contractions, that it does this with no danger to mother or child, that it is chiefly indicated in tedious labors and with primiparæ, for the nervous and the hysteric. Without trying to furnish a definite rule, he mentions that the best time to give the chloral is when the os has dilated and the expulsive pains have commenced. The only objection to giving the drug during the process of dilatation is that the woman must be kept under the influence of the chloral for a long time. For counter indications in its use he refers the reader to the works on therapeutics, but states that a great number of the supposed counter-indications are only imaginary. In general he considers from two to four grammes sufficient, and recommends that it be given either in two doses, with an interval of half an hour, or, for less rapid effect, one gramme every fifteen minutes. When there is any difficulty in giving it by the stomach, it can as well be given by the rectum. He hazards the opinion that chloral may have disinfectant qualities, and believes that much is yet to be learned regarding such qualities in this drug. On the whole, this article of Dr. Chouppe does not contain much that has not already been worked up in other countries, but, as it will insure the knowledge of these facts in France, its publication will be in no way out of place.

Another new remedy has made its appearance, and is now trying its powers on the inmates of some of the Paris hospitals. The last number of the *Journal de Therapeutique* contains a communication from Dr. Henry Blanc, of Bombay, and this article is entitled, "Notes on the External Use of the Powder of Goa for certain Diseases of the Skin." The nature of the powder has not yet been fully decided, for Dr. Blanc writes of it as follows: "It is a vegetable substance, but its origin is still enveloped in mystery; it is fabricated at Goa, and by some it is thought to be from a kind of lichen that is exported in large quantities from Mozambique; others believe, and among them Professors D. S. Kemp and Attfield, that it is from the dry pith of a tree of the Coesalpina order. A large quantity is imported into India, and as only a few grammes are needed to cure an acute attack of herpes circinatus, the natives use a great deal of it." The Doctor narrates his experience with the Powder of Goa in treating "ring-worm," and writes that when all other remedies had failed, he was with this powder able to cure in a few days all the many cases that he had in charge. The manner of applying the substance is very simple. After moistening the diseased surface, the powder is rubbed in by the finger. For internal use an infusion and aqueous extract are made, but Dr. Blanc does not give any details concerning its value when used in this way. Analysis shows that the powder contains a very large quantity of chrysophanic acid. This acid, with certain resins and a bitter principle, are thought to contain the therapeutic principles of rhubarb. From this Dr. Blanc believes that the Powder of Goa may become a rival of that drug. M. Gubler has received a quantity of the powder, and is experimenting with it at the Beaujon hospital. We will have to await the result of his observations.

C. C. MATTESON.

PARIS, June 2, 1875.

